# Cholesterol Crystal Embolization Exacerbates Critical Limb Ischemia

**DOI:** 10.7759/cureus.59498

**Published:** 2024-05-02

**Authors:** Sei Komatsu, Mitsuhiko Takewa, Shigeru Santo, Chikao Yutani, Hirakazu Murayama, Satoru Takahashi, Nobuzo Iwa, Tomoki Ohara, Atsushi Yoshida, Kazuhisa Kodama

**Affiliations:** 1 Department of Cardiology, Osaka Gyoumeikan Hospital, Osaka, JPN; 2 Department of Orthopedics, Osaka Gyoumeikan Hospital, Osaka, JPN; 3 Department of Pathology, Osaka Gyoumeikan Hospital, Osaka, JPN

**Keywords:** endovascular treatment (evt), angioscopy, cholesterol crystals, cholesterol embolization syndrome, chronic life-threatening ischemia

## Abstract

Chronic life-threatening ischemia (CLTI), characterized by chronic severe ischemic ulcers or gangrene in the legs with arterial occlusive disease, has a high rate of amputation and mortality. However, how lower extremity artery disease (LEAD) leads to CLTI is not fully understood yet. Here, we report a 79-year-old man with resting pain and gangrene in the left first and fifth toes for a year who had undergone repetitive endovascular treatment (EVT) that temporarily improved the ischemia. Non-obstructive general angioscopy (NOGA) revealed yellow and red floating emboli at the occluded left superficial femoral artery (SFA). Although a second EVT for the reoccluded SFA was successful, amputation of the left lower knee remained necessary because of osteomyelitis of the left heel. Cholesterol crystals (CCs) associated with innate inflammation were detected in spontaneously ruptured aortic plaques (SRAPs) via aortic screening using the NOGA, in occluded SFAs, and on the surface of the muscle cross-section of the amputated legs via a polarizing microscope. Histopathological analysis demonstrated CCs in small vessels in various stages of patchy necrosis and muscle regeneration. In this case, the process of CC embolization, such as the embolic source of CCs, occlusion in arteries, small arteries, and deposition in muscles, was confirmed in CLTI. CCs are the principal trigger of IL-6 production through the innate inflammatory response in spontaneously ruptured aortic plaques. Mechanical ischemia and chronic inflammation due to embolized CCs may cause chronic limb damage. In this case, the CC embolization might exacerbate CLTI.

## Introduction

Chronic life-threatening ischemia (CLTI) causes an increased risk of cardiovascular events, amputation, and death [1] and is the terminal stage of lower extremity artery disease (LEAD). However, the factors causing LEAD leading to CLTI have yet to be ascertained to date. Atherothromboembolism (ATE) is a common cause of CLTI. [2] Recently, aortic screening with non-obstructive general angioscopy (NOGA) has revealed a high incidence of spontaneously ruptured aortic plaques (SRAPs), including cholesterol crystals (CCs), which are the cause of embolism. Although clinically silent, CCs can cause tissue damage via mechanical injury, ischemia via obstruction, or innate inflammation [3,4]. Free CCs can be dissolved in organic solvents by hematoxylin and eosin staining. In addition to the standard histopathological analysis, we directly scanned the CCs in the blood and muscle samples of the lower extremities using polarized light microscopy [5].

## Case presentation

**Figure 1:**
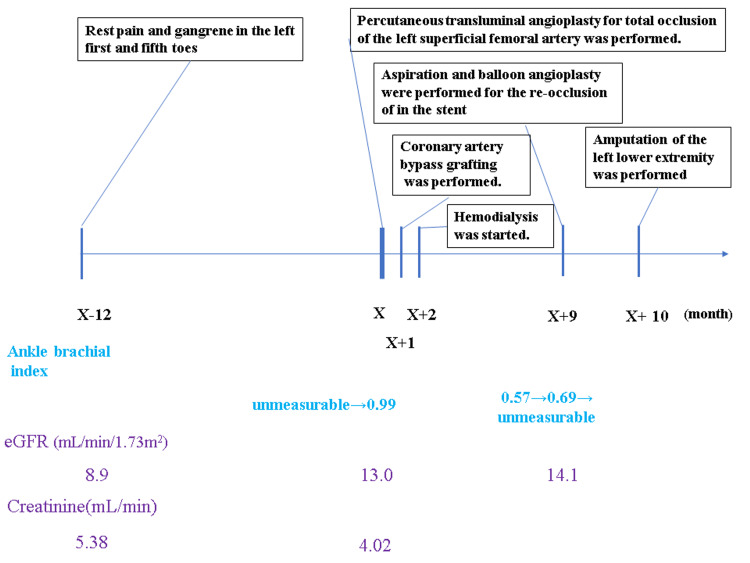
Clinical course of the patient. X: the day of an admission; eGFR: estimated glomerular filtration rate.

A 79-year-old male who was scheduled to undergo chronic hemodialysis was admitted to our hospital with complaints of rest pain and gangrene in the left first and fifth toes for a year (Figure 1).

**Figure 2:**
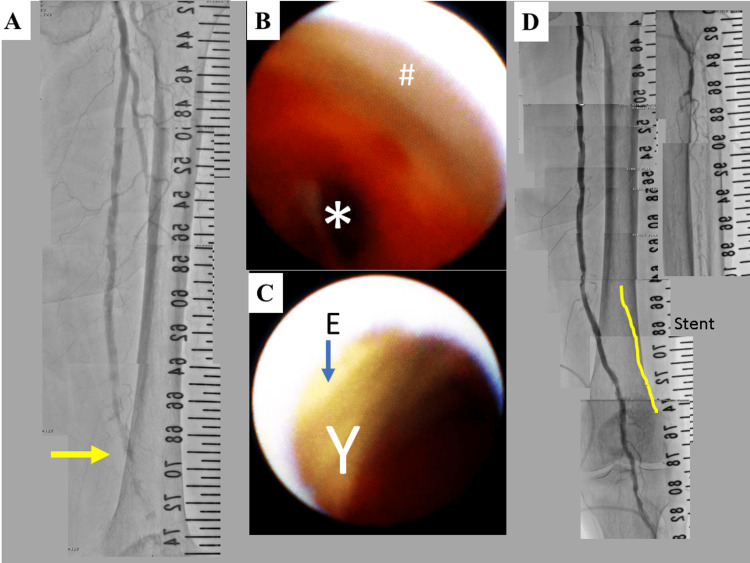
Aortography and angioscopy before and after the first endovascular treatment. (A) Aortography before the endovascular treatment (EVT) on admission. An occlusion is shown in the left superficial femoral artery (SFA). (B) Non-obstructive general angioscopy (NOGA) shows the smooth arterial surface (#) proximal to the occluded site (*) in the left SFA. (C) Yellow (Y) and red floating puff-like emboli (E) in the occluded site shown. (D) Aortography after EVT.

The patient had a history of diabetes, hypertension, chronic renal failure, and cerebral infarction. He did not have a history of smoking. He has been on medication with 100 mg of aspirin, 200 mg of cilostazol, and 15 μg of limaprost alfadex. The blood pressure in his left lower limb was unmeasurable. The WIFi clinical stage was 4 (W-0, Ischemia-3, fi-2). The endovascular treatment (EVT) was performed. An aortography demonstrated an occlusion in the left superficial femoral artery (SFA) (Figure 2A).

Recanalization by NOGA-guided proceeding was successful (Video 1), thereby indicating that the obstruction was due to embolism rather than chronic total occlusion.

**Video 1:** Preoperative angioscopic video of the first and second endovascular treatments and puff chandelier rupture in the infrarenal abdominal aorta.

**Figure 3:**
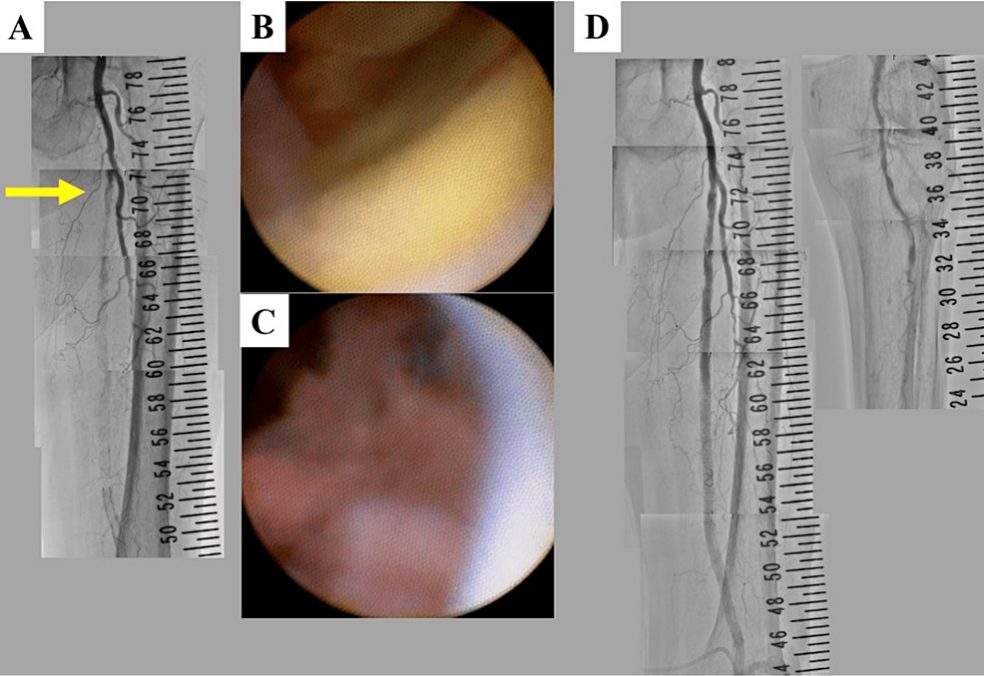
Aortography and angioscopy before and after the second endovascular treatment. (A) Aortography before the second EVT; (B) puff-like emboli in an occlusion site shown via NOGA; (C) red thrombi in the stent shown via NOGA; (D) aortography after the second EVT.

Yellow and red floating emboli were demonstrated using NOGA, although a smooth surface was observed on the proximal inner surface (Figure 2B-2C and Video 1). Recanalization in the SFA with a stent (6 mm × 80 mm ELUVIA: Boston Scientific, MA, USA) was successfully performed (Figure 2D). The right ankle-brachial index (ABI) improved to 0.99. The patient's gangrene was not expanded, and he was free from rest pain in the toes. He underwent minimally invasive coronary artery bypass grafting a month after EVT. The left internal thoracic artery was used to bypass the left anterior descending artery. He also started hemodialysis three times a week, two months after EVT. Nine months after the first EVT, the gangrene worsened. The ABI decreased to 0.57. He underwent the second EVT. Aortography showed re-occlusion inside the stent in the left SFA (Figure 3A).

**Figure 4:**
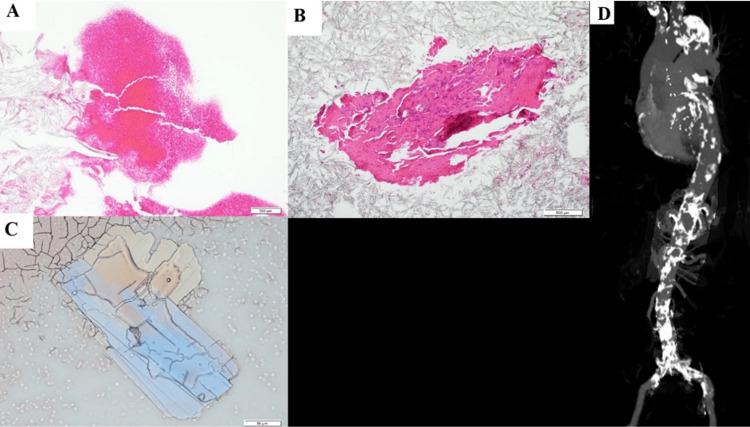
Histopathology obtained during the second endovascular treatment and computed tomography of the aorta. (A) An organized in-stent thrombus stained with hematoxylin-eosin (HE) staining. Bar: 200 μm. (B) A fresh in-stent thrombus stained with HE staining. Bar: 500 μm. (C) Cholesterol crystals are detected in blood samples from the occluded site. Bar: 50 μm. (D) Maximum intensity projection of the computed tomography angiography of the aorta. Severe calcification is distributed in the entire aorta.

NOGA detected puff-like emboli at the occlusion site and red thrombi in the stent (Figure 3B-3C and Video 1). Recanalization after aspiration was successfully achieved with additional stent implantation (6 mm × 40 mm and 7 mm × 120 mm ELUVIA) (Figure 3D). There was no artery below the knee, which was an indication of EVT. Blood samples from spontaneously ruptured aortic plaques were smeared onto 15-cm filter paper with a 7-mm pore size (Advantec Co., Ltd., Tokyo, Japan). Subsequently, a glass plate (40 mm × 22 mm) was placed on the blood and collected using tweezers. If CCs were present, they adhered to the rear surface of the glass. The glass plate was then placed on a powderless glass slide (76 mm × 26 mm) and sealed with resin. The packed glass slides were analyzed within seven days of preparation. Polarized-light microscopy was used to detect the CCs (a touch method). Histopathological analysis revealed organized thrombi in the stent as well as fresh and organized thrombi (Figure 4A-4B).

**Figure 5:**
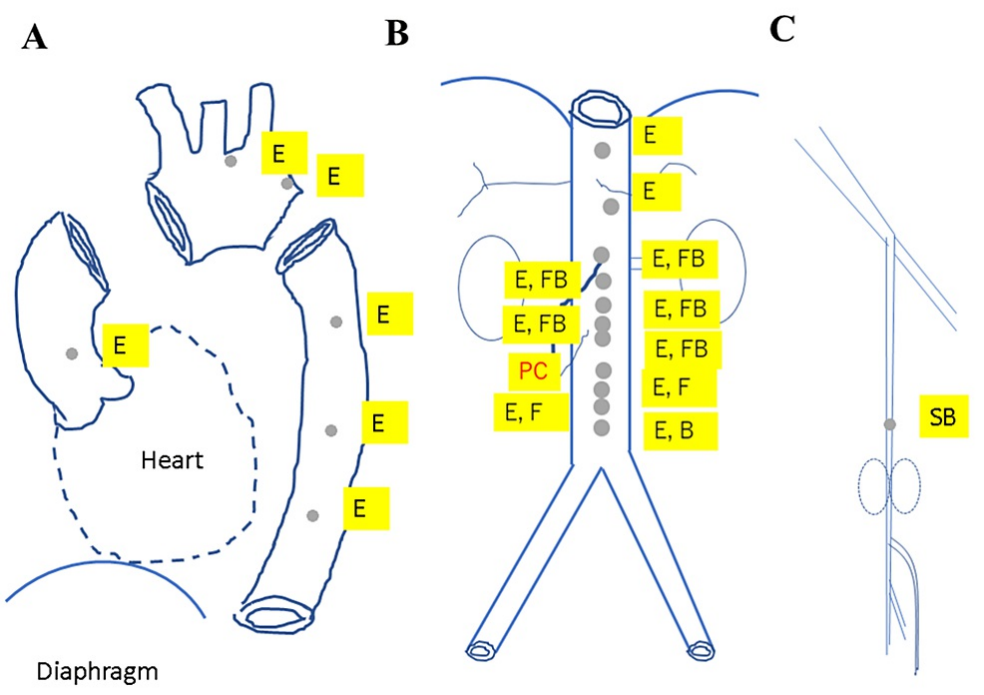
The distribution of spontaneously ruptured aortic plaques in the thoracic (A), the abdominal (B), and the iliac and femoral arteries (C). PC: puff chandelier; E: erosion; F: fissure, FB: fissure bleeding; SB: subintimal bleeding. Image credits: Sei Komatsu.

Using the original touch method, CCs were detected from blood samples of the occluded site (Figure 4C). Computed tomography and angiography showed remarkable calcification in the aorta (Figure 4D). Aortic screening using NOGA demonstrated that SRAPs such as puff-chandelier rupture, erosion, and fissure bleeding were predominantly distributed (Figure 5A-5C).

**Figure 6:**
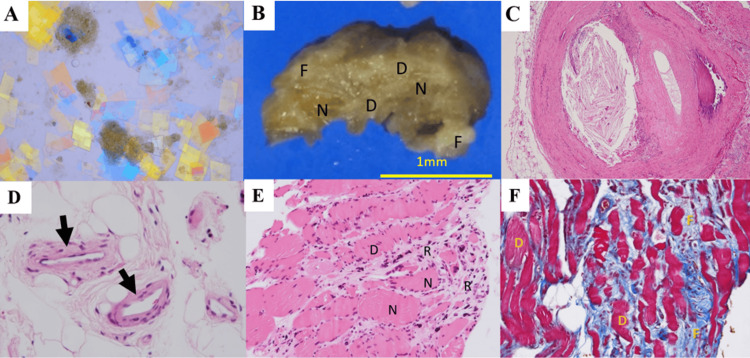
Histopathology of an amputated limb. (A) Cholesterol crystals are directly detected via the stamp method and visualized using polarized light microscopy (×100); (B) macroscopic images of the flexor hallucis longus muscle. N: normal; R: regenerative tissue; F: fibrosis; scale Bar: 1 mm. (C) Hematoxylin-eosin stain of the atheroemboli with CCs in the posterior tibial artery (×100); (D) empty clefts suggest cholesterol crystals in the small arteries (arrows) (×200); (E) histopathological image of the flexor hallucis longus muscle stained using hematoxylin and eosin. N: patchy necrosis, R: regeneration, and D: degeneration (×100). (F) Histopathological image of the flexor hallucis longus muscle stained with Masson’s trichrome. D: patchy degeneration, F: fibrosis (×200).

The estimated number of CCs from a total of 10 mL of blood samples was 4,545. After two sessions of catheterization, no elevation in the eosinophils was observed. The ABI improved to 0.69 on the next day of the second EVT. However, the gangrene worsened again, necrosis of the dorsum of the foot occurred, and osteomyelitis of the left heel was not improved regardless of antibiotics; thus, the left lower extremity was amputated after the second EVT. The amputated limb was inspected to determine the cause of repeated critical ischemia. The surface of the muscle cross-section of the amputated limb was stamped onto a glass slide and observed under a polarizing microscope immediately after the surgery (a stamp method), revealing numerous CCs (Figure 6A).

This suggested that the CCs were distributed in the muscles of the left lower extremity. Macroscopically, we observed sporadic fibrosis and degenerative necrosis (Figure 6B). Histopathological analysis revealed atheroemboli with CCs in the tibial, peroneal, and small arteries (Figure 6C-6D), and various stages of necrosis, regeneration, and degeneration in muscles were found in patches (Figure 6E-6F). SRAPs caused necrosis and muscle regeneration, which worsened the CLTI. He died of intestinal necrosis 18 days after an amputation of his left leg.

## Discussion

In this report of a patient with CLTI, we demonstrated various stages of CC embolism, such as puff-chandelier rupture scattering, emboli in the arteries and muscles, patchy necrosis, and muscle degeneration. This may seem well-known, but this is a new proof of both an embolic source and emboli demonstrated in one patient with CLTI. Our findings suggest a recurrent embolism in the special temporal distribution related to CLTI. SRAPs are found in 80.9% of patients with or suspected of coronary artery disease, and CCs are included in puff-chandelier ruptures among them [3]. CCs can form either as aggregates with atheromatous components or as standalones and may cause organ damage owing to mechanical obstruction and innate inflammation of the small arteries [4]. Many factors, such as oxidized low-density lipoprotein cholesterol, also induce the NLRP3 inflammasome in advanced atherosclerotic plaques. There was a moderate correlation between the counts of CCs and IL-6 ratios in debris from SRAPs sampled by NOGA. CCs are found to be the principal trigger of IL-6 production through the innate inflammatory response among various cascades of plaque progression and rupture in the aorta [5]. The emboli, corresponding to their respective sizes, can cause occlusion in various sizes of arteries. Also, inflamed CCs with inflammatory cytokines may scatter from SRAPs and cause inflammation at embolized sites [4]. Inflammation may cause organ damage in addition to mechanical ischemia. To date, identifying the source of embolism and the disposition of emboli in the muscles of the lower extremities has been challenging due to technical issues. CCs may be mistakenly undetected in standard histopathology staining with hematoxylin and eosin because organic solvents in the preparation may dissolve CCs [6]. NOGA enabled the detection of SRAPs in the aorta, and direct identification of free CCs using polarized light microscopy has further overcome the challenges in detecting CCs. Screening for SRAPs using NOGA can suggest the presence or absence of embolisms in patients with peripheral arterial disease, or CLTI. The effect of lipid-lowering therapy on the decrease in the rate of amputation is controversial [7,8]. If CCs exacerbate LEAD, lipid-lowering therapy and anti-inflammatory agents may be potentially effective. An accumulation of cases is necessary to determine whether this pathophysiology is truly associated with the deterioration of CLTI.

In conclusion, this case report has demonstrated CC embolization in CLTI, encompassing embolic sources, arterial occlusion, and muscle deposition. CC embolization is thought to exacerbate CLTI.

## Conclusions

CCs are the principal trigger of IL-6 production through the innate inflammatory response, which is involved in various cascades of plaque progression and rupture in the aorta. CCs with inflammatory cytokines may scatter from SRAPs and cause inflammation at embolized sites. Mechanical ischemia and chronic inflammation may cause systemic organ damage, such as limb damage. We demonstrated CC embolization in CLTI, encompassing embolic sources, arterial occlusion, and muscle deposition. The CC embolization is thought to exacerbate CLTI in this case. An accumulation of cases is necessary to determine whether the pathophysiology is true.
